# Association between weight control failure and suicidal ideation in overweight and obese adults: a cross-sectional study

**DOI:** 10.1186/s12889-016-2940-1

**Published:** 2016-03-15

**Authors:** Yeong Jun Ju, Kyu-Tae Han, Tae-Hoon Lee, Woorim Kim, Jeong Hun Park, Eun-Cheol Park

**Affiliations:** Department of Public Health, Graduate School, Yonsei University, Seoul, Republic of Korea; Institute of Health Services Research, Yonsei University College of Medicine, Seoul, Republic of Korea; Department of Preventive Medicine, Yonsei University College of Medicine, Seoul, Republic of Korea; Department of Health Administration and Management, College of Medical Science, Soonchunhyang University, Asan, Republic of Korea; Department of Preventive Medicine & Institute of Health Services Research, Yonsei University College of Medicine, 50 Yonsei-ro, Seodaemun-gu, Seoul, 120-752 Korea

**Keywords:** Suicidal ideation, Obesity, Weight control failure, Weight stigma, Middle age

## Abstract

**Background:**

Korea has the highest suicide rate in the OECD and is one of the few OECD countries whose suicide rates have not decreased in recent years. To address these issues, we investigated the effects of weight control failure on suicidal ideation in the overweight and obese populations.

**Methods:**

We performed a cross-sectional study using data from the Korea National Health and Nutrition Examination Survey (2008–2012) consisting of 6621 individuals 40 years of age or older. Logistic regression analysis was used to identify the relationship between weight control failure and suicidal ideation in the overweight and obese populations.

**Results:**

A total of 6621 participants were analyzed in this study (overweight group: 2439; obese group: 4182). Among them, weight control failure (weight gain with weight loss efforts) was experienced in 962 obese (males 16.3 %, females 29.6 %) and 412 overweight individuals (males 9.1 %, females 23.4 %). Weight control failure was significantly associated with suicidal ideation in obese females (OR = 1.70, 95 % CI 1.21–2.39), but this association was not significant in obese males or in either sex of the overweight group.

**Conclusions:**

Findings from this study suggest that weight control failure is associated with an increased risk of suicidal ideation among obese women. Furthermore, intervention programs that aim to address the prevalence of suicide, especially for obese women, are needed.

## Background

Suicide is becoming a public health issue in many countries, and even more so in Korea [1]. South Korea’s suicide rate has been the highest among OECD countries for over a decade. In 2013, the rate of death by intentional self-harm in South Korea was 29.1/100,000 individuals, which was followed by the rates in Japan, Hungary, and Slovenia at nearly 20/100,000 individuals; the OECD average rate was 12/100,000 individuals [2]. Meanwhile, one previous study reported that suicidal ideation is the main thought process leading to suicide [3]. Those who do not envision suicide rarely attempt suicide, whereas 34–42 % of individuals who imagine committing suicide subsequently attempt it [4]. Hence, it is necessary to approach suicide prevention in terms of prevention and identification of the factors that influence suicidal ideation.

Previous studies to clarify the factors affecting suicidal ideation have generally considered relevant health behaviors (drinking, socioeconomic conditions, internet addiction) and mental health conditions (depression, mood disorders, anxiety) [5–7]. However, there remain many issues and debates related to mental health, and suicidal problems in particular, in South Korea. Therefore, we determined it necessary to address suicidal problems from another perspective. In particular, we focused on suicidal problems related to weight control issues in the overweight and obese population of South Korea. However, few studies have examined how weight status impacts suicidal ideation.

Weight status has been classified as underweight, normal weight, overweight or obese. According to a previous study, weight status was associated with major depression, suicide attempts, and suicidal ideation [3]. Obesity is an especially important factor affecting mental health [8, 9]; obese populations have a higher rate of depression and suicidal ideation than that of the normal weight population [10]. In addition, obesity has been associated with increased morbidity and mortality risks [11, 12]. For that reason, many obese individuals attempt to lose weight, and weight reduction is an important aspect of obesity treatment [13]. As a result, weight control is an important issue for the obese population. Given the relationship between obesity and mental health, a study on whether weight control influences suicidal ideation is needed.

Of course, previous studies on suicidal ideation related to weight control exist. However, those studies only focused on adolescents [14–16], while the present study focuses on middle-aged adults. The study was limited to middle-age subjects (40 years or older), because the incidence of many health problems tends to increase during this period [17]. Also, weight status and mental health problems may be different in middle-aged and older adults compared with younger adults, because functional limitations and medical comorbidities related to aging may lead to weight change and mood changes [18]. Especially, women usually experienced the menopause in middle-age, which may leads to health risks such as weight gain [19, 20]. We analyzed the effects of weight control failure on suicidal ideation in the overweight and obese populations and examined the relationship between weigh control failure and suicidal ideation according to income level, household composition, and menopause status.

## Methods

### Study population

**Fig. 1:**
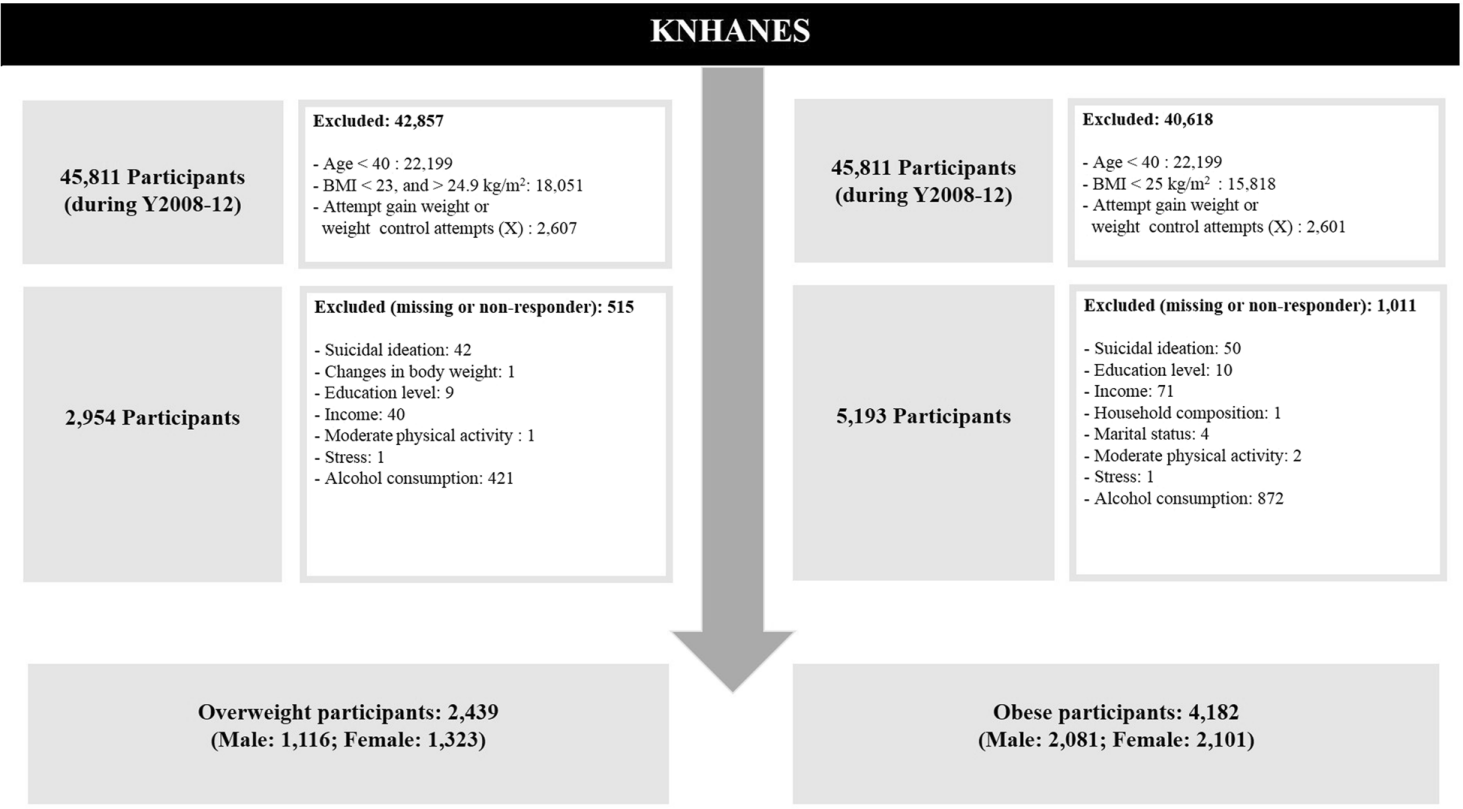
Flow diagram of the study participants

For this cross-sectional study, we used raw data from the Korea National Health and Nutrition Examination Surveys (KNHANES) conducted in 2008–2012 (4th and 5th KNHANES); these are cross-sectional surveys that have been conducted annually by the Korea Centers for Disease Control and Prevention (KCDC). These are cross-sectional surveys with study populations from multistage, stratified area probability samples of civilian non-institutionalized Korean households by geographic area, age, and gender groups. This survey is composed of three parts—a health interview, health examination, and nutrition survey—all of which were performed by trained medical staff and dieticians. We used data from all three parts. These data were collected from a total of 45,811 participants during 2008–2012 (8058 in 2008, 8518 in 2009, 8958 in 2010, 10,533 in 2011, 9744 in 2012). The overall response rates were 74.3 % in 2008, 79.2 % in 2009, 81.9 % in 2010, 80.4 % in 2011, 80.8 % in 2012. In our analysis, the subjects were divided into two subgroups according to BMI: 23–24.9 kg/m^2^ (overweight) or ≥25 kg/m^2^ (obese). Any respondents who did not provide data on BMI, suicidal ideation, education level, income, household composition, marital status, moderate physical activity, stress awareness, alcohol consumption, or who were under the age of 40 years were excluded from the study (see details in Fig. 1). In addition, we excluded those who did not undergo weight control attempts or who attempted to gain weight to investigate the success or failure of weight control efforts in the participants. A total of 6621 eligible participants were included in the present study (2439 overweight; 4182 obese). Meanwhile, considering that sex affects mental health, the present study analyzed men and women separately (overweight: 1116 males, 1323 females; obese: 2081 males, 2101 females) [4, 21]. The KNHANES data are openly available at the KNHANES website: https://knhanes.cdc.go.kr/knhanes/eng/index.do.

Ethical approval for this study was granted by the institutional review board (IRB) of the KCDC Seoul, Korea (IRB #: 2008-04EXP-01-C; 2009-01CON-03-2C; 2010-02CON-21-C; 2011-02CON-06-C; 2012-01EXP-01-2C).

### Measures

#### Suicidal ideation

We used data from a self-reported questionnaire, in which suicidal ideation was assessed by a single item: “Have you ever seriously thought about suicide in the past year?” Responses were rated on a 2-point scale: yes or no. Despite limitations due to the question’s simplicity, previous studies have successfully used this method based on KNHANES data [4, 22, 23].

#### Weight control failure

To assess the success or failure of weight control efforts, we used data from a self-reported questionnaire regarding changes in body weight and weight control efforts. Change in body weight was assessed by the single question, “Have you experienced a change in body weight in comparison with the last year?” Responses were rated on a 3-point scale: weight maintenance, weight gain, or weight loss. Weight gain is composed of three parts: increases of 3–6 kg, 6–10 kg, or 10 kg or more. Weight loss was composed of three parts: decreases of 3–6 kg, 6–10 kg, or 10 kg or more. Weight control efforts were assessed by the single question, “Have you ever tried to control your weight voluntarily in the last year?” Responses were rated on a 4-point scale: made efforts for weight loss, made efforts for weight maintenance, made efforts for weight gain, did not make efforts for weight control. Finally, we classified weight control failure as “yes” or “no”. Those who experienced weight gain during weight loss efforts were categorized as “yes”. Those who experienced weight loss during weight loss efforts or weight maintenance during weight maintenance efforts were classified as “no”.

#### Criteria of obesity

The World Health Organization Regional Office for the Western Pacific Region recommends defining obesity in Asians as those with a BMI ≥25 kg/m^2^. Korea officially uses this definition when calculating the prevalence of obesity in Korea. Overweight in Asians is defined as a BMI of 23–24.9 kg/m^2^ [24]. Therefore, our study used this definition.

#### Covariates

We included age, sex, income, educational level, economic activity, marital status, household composition, smoking status, alcohol consumption, stress awareness, depressive symptoms, perceived body image, perceived health status, moderate physical activity, menopause (only for females) and survey year as covariates. Age was categorized into four groups, starting with 40 years of age. Income status was classified as “low”, “middle”, or “high”. Economic activity was classified as “employed” or “unemployed”. Household composition was assessed by the question “What is your household composition?” This variable was categorized as “one-generation household” (single-person household), “two-generation household” (couple + children, single parent + children, couple + parents, couple + single parent, grandparents + grandchildren, single grandparent + grandchildren), and “three-generation household” (couple + parents + children, couple + single parent + children). Stress awareness was classified as “high” or “low”. Depressive mood was assessed by duration of the depressive mood during the past 2 weeks (present, absent). Subjective body perception was assessed by the question “How would you categorize your current body shape?” (very thin, slightly thin, normal, slightly fat, or very fat). We categorized this as “slim and normal” (very thin, slightly thin, or normal) or “fat” (slightly fat or very fat). Moderate physical activity was classified as whether respondents performed moderate physical activity for 30 min per session more than five times per week. Menopause status was assessed as “yes” (experienced menopause) or “no” (not applicable or yet to experience menopause).

### Statistical analysis

We first examined the distribution of each categorical variable. The Chi-squared test was used to calculate the frequencies and percentages of the variables and to identify significant differences between groups. The analyses for incidence of suicidal ideation by sex were also performed. In addition, to produce an unbiased national estimate, a sample weight was assigned for the participating individuals to represent the Korean population. The sampling weight were calculated by accounting for the complex survey design, survey nonresponse, and post-stratification [25]. Next, multivariable logistic regression analysis was used to examine the association between weight control failure and suicidal ideation while controlling for potential confounding variables such as age, sex, income, educational level, economic activity, marital status, household composition, alcohol consumption, stress awareness, depression mood, subjective body image, perceive health status, moderate physical activity, menopause (only females) and survey year. In addition, the fit of the model was assessed using the Hosmer-Lemeshow goodness-of-fit test; well-fitting logistic models have a nonsignificant goodness-of-fit [26]. Multicollinearity was tested using a variance inflation factor (VIF), which provided an index to measure how much the variance of an estimated regression coefficient increased due to collinearity. If the value of the variance inflation factor exceeded 10, a model was regarded as indicating collinearity. Finally, subgroup analyses were performed to evaluate the association between weight control failure and suicidal ideation according to income, household composition, or menopause. All of the analyses were performed using SAS 9.4 software (SAS Institute, Cary, NC, USA) and the statistical significance level was set at *p*-value < 0.05.

## Results

In our study, a total of 6621 participants were included to assess the association between weight control failure and suicidal ideation (overweight: 2439; obese: 4182). Tables 1 and 2 show the characteristics of the study population by sex. Among the obese population, suicidal ideation was higher in females than males. Suicidal ideation was noted in 195 (9.4 %) males and 432 (20.6 %) females. Similar trends were demonstrated in the overweight population (10.0 % in males, 17.5 % in females). Weight control failure was more frequent in the obese group. Among the obese, weight control failure was higher in females than males (16.3 % in males, 29.6 % in females). Among the overweight, 9.1 % of males versus 23.4 % of females reported that they experienced weight control failure. Notably, subjects in both the obese and overweight populations who experienced weight control failure were more likely to report suicidal thoughts. Regarding subjective body perception, among overweight males, 379 (34.0 %) reported being fat, whereas obese males reported being fat (*n* = 1656; 79.6 %). Conversely, most females responded as being fat weight regardless of weight status (64.7 % in overweight, 88.9 % in obese).

**Table 1:** General characteristics of participants according to suicidal ideation in male

Variables	Suicidal ideation (Male)					
	BMI 23 ~ 24.9			BMI ≥25		
	Total	Yes		Total	Yes	
	N (%)	N (%)	*P*-value*	N (%)	N (%)	*P*-value*
Weight control failure			0.7918			0.6632
No	1014 (90.9)	101 (10.0)		1741 (83.7)	161 (9.3)	
Yes	102 (9.1)	11 (10.8)		340 (16.3)	34 (10.0)	
Age			0.7125			0.0053
40 ~ 49	335 (30.0)	29 (8.6)		760 (36.5)	56 (7.4)	
50 ~ 59	365 (32.7)	40 (10.9)		612 (29.4)	59 (9.6)	
60 ~ 69	276 (24.7)	27 (9.8)		478 (23.0)	45 (9.4)	
≥70	140 (12.6)	16 (11.4)		231 (11.1)	35 (15.1)	
Education level			0.0523			<.0001
Less than middle school	346 (31.0)	46 (13.3)		659 (31.7)	91 (13.8)	
High school graduate	368 (33.0)	32 (8.7)		697 (33.5)	62 (8.9)	
University graduate	402 (36.0)	34 (8.5)		725 (34.8)	42 (5.8)	
Income			0.0021			<.0001
High	370 (33.2)	23 (6.2)		643 (30.9)	40 (6.2)	
Middle	535 (47.9)	57 (10.6)		1030 (49.5)	89 (8.6)	
Low	211 (18.9)	32 (15.2)		408 (19.6)	66 (16.2)	
Economic activity			0.0035			0.0012
Employed	843 (75.5)	72 (8.5)		1658 (79.7)	138 (8.3)	
Unemployed	273 (24.5)	40 (14.6)		423 (20.3)	57 (13.5)	
Marital status			0.9428			0.0297
Married	1097 (98.3)	110 (10.0)		2039 (98.0)	187 (9.2)	
Single	19 (1.7)	2 (10.5)		42 (2.0)	8 (19.0)	
Household composition			0.0936			0.1951
First Generation	354 (31.7)	34 (9.6)		635 (30.5)	67 (10.5)	
Two Generation	624 (55.9)	57 (9.1)		1203 (57.8)	112 (9.31)	
Three Generation	138 (12.4)	21 (15.2)		243 (11.7)	16 (6.6)	
Stress awareness			<.0001			<.0001
Low	885 (79.3)	51 (5.8)		1610 (77.4)	92 (5.7)	
High	231 (20.7)	61 (26.4)		471 (22.6)	103 (21.9)	
Depression mood			<.0001			<.0001
Absent	1015 (91.0)	60 (5.9)		1895 (91.1)	105 (5.5)	
Present	101 (9.0)	52 (51.5)		186 (8.9)	90 (48.4)	
Alcohol intake			0.4252			0.2811
No	152 (13.6)	18 (11.8)		259 (12.5)	29 (11.2)	
Yes	964 (86.4)	94 (9.7)		1822 (87.5)	166 (9.1)	
Moderate physical activity			0.3373			0.1114
No	586 (52.5)	54 (9.2)		1061 (51.0)	110 (10.4)	
Yes	530 (47.5)	58 (10.9)		1020 (49.0)	85 (8.3)	
Subjective body perception			0.6795			0.2492
Slim & Normal	737 (66.0)	72 (9.8)		425 (20.4)	46 (10.8)	
Fat	379 (34.0)	40 (10.5)		1656 (79.6)	149 (9.0)	
Perceive health status			<.0001			<.0001
Healthy	951 (85.2)	80 (8.4)		1750 (84.1)	118 (6.7)	
Unhealthy	165 (14.8)	32 (19.4)		331 (15.9)	77 (23.3)	
Year			0.0942			0.1057
2008	187 (16.8)	19 (10.2)		364 (17.5)	42 (11.5)	
2009	254 (22.8)	35 (13.8)		483 (23.2)	32 (6.6)	
2010	203 (18.2)	14 (6.9)		414 (19.9)	38 (9.2)	
2011	236 (21.1)	26 (11.0)		438 (21.0)	48 (11.0)	
2012	236 (21.1)	18 (7.6)		382 (18.4)	35 (9.2)	
Total	1116 (100.0)	112 (10.0)		2081 (100.0)	195 (9.4)	

Notes: * *P*-values calculated by the chi-square test

**Table 2:** General characteristics of participants according to suicidal ideation in female

Variables	Suicidal ideation (Female)					
	BMI 23 ~ 24.9			BMI ≥25		
	Total	Yes		Total	Yes	
	N (%)	N (%)	*P*-value*	N (%)	N (%)	*P*-value*
Weight control failure			0.1292			0.0239
No	1013 (76.6)	168 (16.6)		1479 (70.4)	285 (19.3)	
Yes	310 (23.4)	63 (20.3)		622 (29.6)	147 (23.6)	
Age			0.1454			<.0001
40 ~ 49	523 (39.5)	76 (14.5)		648 (30.8)	107 (16.5)	
50 ~ 59	472 (35.7)	92 (19.5)		696 (33.1)	127 (18.2)	
60 ~ 69	267 (20.2)	50 (18.7)		530 (25.2)	32 (24.9)	
≥70	61 (4.6)	13 (21.3)		227 (10.9)	66 (29.1)	
Education level			<.0001			<.0001
Less than middle school	613 (46.3)	138 (22.5)		1275 (60.7)	310 (24.3)	
High school graduate	487 (36.8)	68 (14.0)		613 (29.2)	90 (14.7)	
University graduate	223 (16.9)	25 (11.2)		213 (10.1)	32 (15.0)	
Income			<.0001			<.0001
High	393 (29.7)	52 (13.2)		479 (22.8)	74 (15.4)	
Middle	662 (50.0)	106 (16.0)		1112 (52.9)	221 (19.9)	
Low	268 (20.3)	73 (27.2)		510 (24.3)	137 (26.9)	
Economic activity			0.4947			0.0074
Employed	657 (49.7)	110 (16.7)		1030 (49.0)	187 (18.2)	
Unemployed	666 (50.3)	121 (18.2)		1071 (51.0)	245 (22.9)	
Marital status			0.5919			0.6217
Married	1310 (99.0)	228 (17.4)		2081 (99.0)	427 (20.5)	
Single	13 (1.0)	3 (23.1)		20 (1.0)	5 (25.0)	
Household composition			0.0925			0.0049
First Generation	391 (29.5)	82 (21.0)		750 (35.7)	169 (22.5)	
Two Generation	779 (58.9)	125 (16.0)		1045 (49.7)	186 (17.8)	
Three Generation	153 (11.6)	24 (16.0)		306 (14.6)	77 (25.2)	
Stress awareness			<.0001			<.0001
Low	996 (75.3)	96 (9.6)		1503 (71.5)	179 (11.9)	
High	327 (24.7)	135 (41.3)		598 (28.5)	253 (42.3)	
Depression mood			<.0001			<.0001
Absent	1091 (82.5)	108 (9.90)		1691 (80.5)	203 (12.0)	
Present	232 (17.5)	123 (53.0)		410 (19.5)	229 (56.0)	
Alcohol intake			0.7716			0.1623
No	294 (22.2)	53 (18.0)		468 (22.3)	107 (22.9)	
Yes	1029 (77.8)	178 (17.3)		1633 (77.7)	325 (19.9)	
Moderate physical activity			0.6327			0.8825
No	789 (59.6)	141 (17.9)		1271 (60.5)	260 (20.5)	
Yes	534 (40.4)	90 (16.9)		830 (39.5)	172 (20.7)	
Subjective body perception			0.1517			0.3125
Slim & Normal	467 (35.3)	91 (19.5)		234 (11.1)	54 (23.1)	
Fat	856 (64.7)	140 (16.4)		1867 (88.9)	378 (20.3)	
Perceive health status			<.0001			<.0001
Healthy	1052 (79.5)	135 (12.8)		1473 (70.0)	226 (15.3)	
Unhealthy	271 (20.5)	96 (35.4)		628 (30.0)	206 (32.8)	
Menopause			0.0891			0.0008
Not yet	588 (44.4)	91 (15.5)		793 (37.7)	133 (16.8)	
Yes	735 (55.6)	140 (19.0)		1308 (62.3)	299 (22.9)	
Year			0.0625			0.0014
2008	246 (18.6)	42 (17.1)		384 (18.3)	92 (24.0)	
2009	285 (21.5)	59 (20.7)		476 (22.7)	115 (24.2)	
2010	258 (19.5)	48 (18.6)		383 (18.2)	77 (20.1)	
2011	268 (20.3)	42 (15.7)		436 (20.8)	76 (17.4)	
2012	266 (20.1)	40 (15.0)		422 (20.1)	72 (17.1)	
Total	1323 (100.0)	231 (17.5)		2101 (100.0)	432 (20.6)	

Notes: * *P*-values calculated by the chi-square test

Table 3 shows the results of the logistic regression analysis for the association between weight control failure and suicidal ideation after multivariable adjustment. Weight control failure was significantly associated with suicidal ideation in the obese females. Among obese females, those with weight control failure were at 1.70-fold higher risk of suicidal ideation compared with those with weight control success or maintenance (OR = 1.70, 95 % CI 1.21–2.39), but this association was not significant in males of the obese or overweight group. In addition, among obese females, those with a low-income status were at a 2.11-fold higher risk of suicidal ideation compared with those with a mid- to high-income status (OR = 2.12, 95 % CI 1.35–3.32); similar trends were evident in obese males (OR = 1.79, 95 % CI 1.02–3.14). Obese females at menopause were at a 1.65-fold higher risk of suicidal ideation compared with obese females who had not yet reached menopause (OR = 1.65, 95 % CI 1.02–2.65), whereas this association was not significant in overweight females. Stress awareness and depression mood were associated with suicidal ideation among all participants. The Hosmer-Lemeshow test was non-significant (*P* = 0.4263 in overweight male group, *P* = 0.3487 in overweight female group, *P* = 0.3573 obese male group and *P* = 0.4730 in obese female group) indicating adequate goodness-of-fit. In addition, All VIFs are below 2, indicating a lack of multicollinearity. The maximum VIF identified in our models was 1.83 (for obese male – age group).

**Table 3:** Results of multivariable logistic regression analysis for the association between weight control failure and suicidal ideation

Variables	Suicidal ideation (Male)								Suicidal ideation (Female)							
	BMI 23 ~ 24.9				BMI ≥25				BMI 23 ~ 24.9				BMI ≥25			
	a-OR^a^	95 % CI			a-OR^a^	95 % CI			a-OR^a^	95 % CI			a-OR^a^	95 % CI		
Weight control failure																
No	1.00	-		-	1.00	-		-	1.00	-		-	1.00	-		-
Yes	0.77	0.33	-	1.80	1.54	0.91	-	2.60	1.09	0.70	-	1.18	1.70	1.21	-	2.39
Age																
40 ~ 49	1.00	-		-	1.00	-		-	1.00	-		-	1.00	-		-
50 ~ 59	1.37	0.64	-	2.94	0.99	0.52	-	1.86	1.19	0.68	-	2.10	0.70	0.42	-	1.17
60 ~ 69	1.13	0.42	-	3.03	1.28	0.54	-	3.04	1.27	0.57	-	2.83	1.14	0.61	-	2.14
≥70	2.27	0.67	-	7.75	1.45	0.59	-	3.60	1.16	0.37	-	3.59	1.43	0.67	-	3.05
Income																
High	1.00		-		1.00		-		1.00		-		1.00		-	
Middle	0.95	0.46	-	1.93	1.02	0.61	-	1.71	0.86	0.52	-	1.43	1.57	1.05	-	2.35
Low	1.52	0.63	-	3.69	1.79	1.02	-	3.14	1.57	0.86	-	2.89	2.12	1.35	-	3.32
Education level																
Less than middle school	1.00	-		-	1.00	-		-	1.00	-		-	1.00	-		-
High school graduate	0.81	0.37	-	1.75	0.56	0.30	-	1.02	0.77	0.47	-	1.26	0.72	0.48	-	1.08
University graduate	0.88	0.38	-	2.01	0.55	0.29	-	1.07	0.60	0.30	-	1.22	0.75	0.42	-	1.35
Economic activity																
Employed	1.00	-		-	1.00	-		-	1.00	-		-	1.00	-		-
Unemployed	1.23	0.64	-	2.41	1.18	0.61	-	2.27	1.29	0.83	-	2.01	0.95	0.70	-	1.31
Marital status																
Married	1.00	-		-	1.00	-		-	1.00	-		-	1.00	-		-
Single	0.51	0.08	-	3.36	3.06	1.00	-	9.34	2.00	0.52	-	7.66	0.30	0.04	-	1.99
Household composition																
Two Generation	1.00	-		-	1.00	-		-	1.00	-		-	1.00	-		-
First Generation	0.94	0.45	-	1.99	0.84	0.45	-	1.56	1.64	0.97	-	2.80	0.93	0.63	-	1.39
Three Generation	1.33	0.58	-	3.05	0.56	0.25	-	1.28	0.87	0.45	-	1.70	1.22	0.79	-	1.89
Alcohol intake																
No	1.00	-		-	1.00	-		-	1.00	-		-	1.00	-		-
Yes	1.17	0.56	-	2.46	0.84	0.45	-	1.56	0.95	0.59	-	1.52	1.08	0.73	-	1.60
Stress awareness																
Low	1.00	-		-	1.00	-		-	1.00	-		-	1.00	-		-
High	3.93	2.24	-	6.90	3.79	2.14	-	4.42	4.78	3.09	-	7.40	3.35	2.45	-	4.59
Depression mood																
No	1.00	-		-	1.00	-		-	1.00	-		-	1.00	-		-
Yes	15.08	8.20	-	27.75	10.35	6.35	-	16.84	6.23	4.10	-	9.49	6.86	4.91	-	9.59
Moderate physical activity																
No	1.00	-		-	1.00	-		-	1.00	-		-	1.00	-		-
Yes	1.28	0.55	-	1.47	0.90	0.55	-	1.47	1.04	0.69	-	1.56	0.88	0.63	-	1.21
Subjective body perception																
Slim & Normal	1.00	-		-	1.00	-		-	1.00	-		-	1.00	-		-
Fat	1.49	0.56	-	2.46	0.71	0.38	-	1.30	0.97	0.62	-	1.50	0.89	0.54	-	1.46
Perceive health status																
Healthy	1.00	-		-	1.00	-		-	1.00	-		-	1.00	-		-
Unhealthy	1.29	0.71	-	2.37	3.14	1.92	-	5.13	2.29	1.42	-	3.71	1.38	1.00	-	1.92
Menopause																
Not yet	-		-		-		-		1.00	-		-	1.00	-		-
Yes	-		-		-		-		1.24	0.73	-	2.11	1.65	1.02	-	2.65
Year																
2008	1.00				1.00				1.00	-		-	1.00	-		-
2009	1.35	0.54	-	3.33	0.39	0.20	-	0.77	0.70	0.35	-	1.40	0.90	0.58	-	1.41
2010	0.95	0.40	-	2.26	0.63	0.33	-	1.22	1.14	0.61	-	2.13	0.91	0.55	-	1.52
2011	1.31	0.50	-	3.41	1.20	0.67	-	2.17	0.97	0.51	-	1.84	0.51	0.31	-	0.83
2012	1.25	0.47	-	3.31	0.86	0.41	-	1.82	0.84	0.43	-	1.64	0.64	0.39	-	1.08
Hosmer-Lemeshow test		*P* = 0.4263				*P* = 0.3487				*P* = 0.3573				*P* = 0.4730		

Notes: ^a^a-OR adjusted by weight control failure, age, income, education level, economic activity, marital status, household composition, alcohol intake, stress awareness, depression mood, moderate physical activity, subjective body perception, perceive health status, menopause, year

The subgroup analysis results are shown in Table 4. Subgroup analysis showed significant differences in each group, even though modifying effects were not significant in the tests for interaction. Among obese males and females, those who had experienced weight control failure showed a trend towards a greater magnitude of suicidal ideation if they were of low, but not high, income status. Among obese females, there was a trend towards a greater magnitude of suicidal ideation if they lived on their own or in a two-generation household, but not if they lived with more family members. Among obese females experiencing menopause, those who had experienced weight control failure showed a trend towards a greater magnitude of suicidal ideation.

**Table 4:** Results of subgroup analysis for the relationship between weight control failure and suicidal ideation by income, household composition, or menopause

Variables		Male								Female							
		BMI 23 ~ 24.9				BMI ≥25				BMI 23 ~ 24.9				BMI ≥25			
		a-OR^a^	95 % CI			a-OR^a^	95 % CI			a-OR^a^	95 % CI			a-OR^a^	95 % CI		
Income	Weight control failure																
High	No	1.00				1.00				1.00				1.00			
	Yes	0.33	0.07	-	1.54	0.52	0.13	-	2.11	0.80	0.31	-	2.03	1.65	0.65	-	4.24
Middle	No	1.00				1.00								1.00			
	Yes	1.86	0.69	-	5.03	1.37	0.66	-	2.88	1.17	0.61	-	2.23	1.67	1.08	-	2.60
Low	No	1.00				1.00								1.00			
	Yes	0.22	0.02	-	2.74	2.27	0.89	-	5.81	1.10	0.42	-	2.90	1.87	1.00	-	3.72
Household composition	Weight control failure																
One generation	No	1.00				1.00				1.00				1.00			
	Yes	0.63	0.13	-	3.03	0.62	0.20	-	1.95	0.74	0.28	-	1.99	1.88	1.08	-	3.25
Two generation	No	1.00				1.00				1.00				1.00			
	Yes	1.29	0.52	-	3.17	2.46	1.32	-	4.57	1.19	0.66	-	2.13	1.64	1.02	-	2.62
Three generation	No	1.00				1.00				1.00				1.00			
	Yes	0.12	0.01	-	1.74	2.26	0.29	-	17.60	2.01	0.22	-	18.1	1.05	0.42	-	2.62
Menopause	Weight control failure																
Not yet	No	-	-		-	-	-		-	1.00				1.00			
	Yes	-	-		-	-	-		-	1.31	0.68	-	2.53	1.58	0.90	-	2.80
Yes	No	-	-		-	-	-		-	1.00				1.00			
	Yes	-	-		-	-	-		-	0.91	0.47	-	1.76	1.80	1.18	-	2.75

Notes: ^a^a-OR adjusted by weight control failure, age, income, education level, economic activity, marital status, household composition, alcohol intake, stress awareness, depression mood, moderate physical activity, subjective body perception, perceive health status, menopause, year

## Discussion

South Korea currently has many public health issues. Among OECD members, Korea has ranked first in suicide rate for 11 years. The mortality rate from suicide increased rapidly from 22.6 % in 2003 to 28.5 % in 2013, making suicide the fourth leading cause of death in Korea [27]. In addition, the prevalence of obesity has continued to increase over the last 10 years, resulting in lifestyle changes. Obesity has become a serious national problem and is no longer a concern only in Western countries [28, 29]. Considering that these issues remain a concern and that obesity is an important factor affecting mental health, it is necessary to design effective strategies to prevent and manage suicidal ideation among the overweight and obese populations.

We found that weight control failure was significantly associated with suicidal ideation among obese females, whereas this association was not significant in obese males or overweight populations after multivariable adjustment. This finding can be explained by weight stigma. Weight stigma has been described as negative weight-related attitudes and beliefs. Obese individuals are often highly stigmatized [30], and obese females experience weight stigma more than do obese males [31, 32]. Weight stigma experiences were significantly related to depressive symptoms, decreased self-esteem and suicidality [33, 34]. Obese females in particular may be more vulnerable to the societal standards of beauty and obesity stigma compared with males, thereby degrading mental health. Hence, weight control failure among obese females may affect experience with weight stigma, thereby deteriorating psychological wellbeing, and in particular, suicidal ideation.

Our findings suggests that efforts to reduce the high suicide rate should target obese females. Considering that the prevalence of obesity is high among middle-aged women and that the age-specific obesity rate in Korean females has recently increased sharply, obese females are an important target group for intervention [35]. Despite its issues remain a concern, programs to resolve issues are rare. From our findings, therefore, we suggest that suicide prevention programs need to focus on supporting obese females, such as encouraging physical activity, and supporting enrollment in weight control programs. In Korea, most weight control programs for obesity are focused on children. Despite positive outcomes, the few programs focusing on obese females experienced limitations such as barriers in the recruitment and retention of participants [36]. Therefore, policy makers should develop strategies for participants’ continuous participation using effectiveness tools, such as telephone counseling and mobile phone SMS messages [37]. Such support can improve not only the mental but also the physical health of obese females.

In addition, our subgroup analysis indicated that menopause, household composition, or low income potentially affect the association between weight control failure and suicidal ideation, even though the modifying effect was not significant. The overall trends seen among our findings have serious implications for the management of suicidal ideation. Several studies offer potential explanations. Regarding menopause, females may experience weight gain during the peri-menopausal to post-menopausal period, and this weight gain may exacerbate the changes in health risk factors that appear during menopause [20, 38]. For that reasons, obese females may experience the weight gain. Thus, policy makers should consider health policy providing hormone injections for women experiencing menopause. Regarding household composition, previous studies reported that family members and support networks including parents, spouses, and friends can increase the effectiveness of weight control in obese populations. Such support networks would be most effective in eradicating negative attitudes and bias regarding weight [39, 40]. Therefore, policy makers should consider programs utilizing family and a support network, it could help weight control in obese populations.

Meanwhile, we found a gradient in suicidal ideation by socio-demographic factors such as education, income, and employment status in both males and females. More disadvantage males and females were more likely to report suicidal ideation, in particular obese females. Few studies offer potential explanations by focusing relationship between socioeconomic gradient and obesity. Which relationship was well established through previous studies [41, 42]. For example, because higher socioeconomic groups tend to have a healthier diet, higher educational attainment, income and occupational status were associated with lower risk of obesity. In addition, studies suggests that the relation between socio-economic inequality and obesity is stronger among females than among males [43, 44]. On the other hands, obese females are socially and economically disadvantaged [45]. They are less likely to practice the healthy dieting thereby increasing the weight. Therefore, weight gain and unhealthy dietary practices due to socio and economic disadvantage in women may lead to more weight control failure and weight stigma, thereby exacerbating the mental health. Meanwhile, few studies has shown an inverse results between socioeconomic status and obesity [46, 47]. These studies found that high education attainment or high income was related to prevalence of obesity in females. Based our results and previous studies, the relationship between social gradient and suicidal ideation among obese population are not currently, because studies on those relationship is less well established. Therefore, further studies on those findings are needed.

This study has several strengths compared with previous studies. First, the study used a large representative sample, and data were collected from a nationally representative population. Second, to our knowledge, our study is the first to report on the relationship between weight control failure and suicidal ideation among obese populations. Previous studies focused only on the relationship between weight-based stigmatization or obesity and mental health in adolescents. Finally, our study focused on the cause of suicidal ideation among overweight and obese populations.

However, there were also several limitations. First, the present study was unable to identify a causal relationship between weight control failure and suicidal ideation, because the study design was cross sectional, and information was obtained via self-report. Second, our subgroup analysis findings are limited with regard to the interpretation of the results, because the modifying effect was not significant, although the findings showed significant differences in each group. Therefore, other studies are needed to confirm our findings. Third, we did not investigate the reasons behind weight control failure and change in weight. Furthermore, we did not consider various factors related to weight change, such as food intake volume, exercise frequency and exercise intensity. Fourth, we could not accurately measure suicidal ideation, because the question pertaining to suicidal ideation required only a “yes” or “no” answer. In addition, we could not accurately assess weight control failure, because the answers were subjective. Therefore, self-reporting of the respondents could have led to an underestimation of the actual relationship between weight control failure and suicidal ideation.

Despite the limitations, this is the first study to investigate the association between weight control failure and suicidal ideation among obese and overweight Koreans. Considering that the high prevalence of suicide and the increasing prevalence of obesity in Korea, our findings are important for health policy makers to identify solutions for controlling suicide problem.

## Conclusion

In conclusion, weight control failure had an effect on suicidal ideation. Suicidal ideation in obese females was significantly higher than that in other populations. Our findings suggest a need to focus on suicidal ideation in middle-aged and older obese females in particular.
